# Titanium trisulfide (TiS_3_): a 2D semiconductor with quasi-1D optical and electronic properties

**DOI:** 10.1038/srep22214

**Published:** 2016-03-02

**Authors:** Joshua O. Island, Robert Biele, Mariam Barawi, José M. Clamagirand, José R. Ares, Carlos Sánchez, Herre S. J. van der Zant, Isabel J. Ferrer, Roberto D’Agosta, Andres Castellanos-Gomez

**Affiliations:** 1 Kavli Institute of Nanoscience, Delft University of Technology, Lorentzweg 1, 2628 CJ Delft, The Netherlands; 2 Nano-Bio Spectroscopy Group and European Theoretical Spectroscopy Facility (ETSF), Universidad del Pais Vasco, 20018 San Sebastian, Spain; 3 Materials of Interest in Renewable Energies Group (MIRE Group), Dpto. de Física de Materiales, Universidad Autónoma de Madrid, 28049 Madrid, Spain; 4 Inst. Nicolas Cabrera, Univ. Autónoma de Madrid, 28049 Madrid, Spain; 5 IKERBASQUE, Basque Foundation for Science, 48013 Bilbao, Spain; 6 Instituto Madrileño de Estudios Avanzados en Nanociencia (IMDEA Nanociencia), Campus de Cantoblanco, 28049 Madrid, Spain

## Abstract

We present characterizations of few-layer titanium trisulfide (TiS_3_) flakes which, due to their reduced in-plane structural symmetry, display strong anisotropy in their electrical and optical properties. Exfoliated few-layer flakes show marked anisotropy of their in-plane mobilities reaching ratios as high as 7.6 at low temperatures. Based on the preferential growth axis of TiS_3_ nanoribbons, we develop a simple method to identify the in-plane crystalline axes of exfoliated few-layer flakes through angle resolved polarization Raman spectroscopy. Optical transmission measurements show that TiS_3_ flakes display strong linear dichroism with a magnitude (transmission ratios up to 30) much greater than that observed for other anisotropic two-dimensional (2D) materials. Finally, we calculate the absorption and transmittance spectra of TiS_3_ in the random-phase-approximation (RPA) and find that the calculations are in qualitative agreement with the observed experimental optical transmittance.

The isolation of graphene and similar atomically-thin, van der Waals materials has sparked a strong research focus on this broad family which can be exfoliated from bulk layered crystals^1^,^2^,^3^. Despite this growing interest, the research focus has been mainly limited to graphene, boron nitride, and the Mo- and W- based transition metal dichalcogenides^4^,^5^,^6^,^7^. These materials, although very different, have largely isotropic in-plane optical and electrical properties. Reduced in-plane symmetry could lead to interesting anisotropic properties furthering the functionalities and applications of two-dimensional (2D) materials. In particular, anisotropic 2D materials would be appealing to fabricate passive optical polarizers and high mobility transistors that benefit from reduced backscattering from hot electrons^8^.

Recently, the layered materials black phosphorus (BP) and rhenium disulfide (ReS_2_) have shown promising in-plane anisotropy of their electrical and optical properties^9^,^10^,^11^. Harnessing the anisotropic properties of these materials has resulted in applications such as an integrated digital inverter^12^, and a linear-dichroic photodetector^13^. Exploration of other layered materials with stronger structural in-plane anisotropy would allow the opportunity to couple the advantages of 2D materials (flexibility, transparency, and large surface to volume ratio) with fully quasi-one dimensional (1D) properties. The transition metal trichalcogenides (with general formula MX_3_) are a prospective family of materials to achieve this goal due to their reduced in-plane bonding symmetry^14^. Fig. 1 shows the crystal structure of a single layer of titanium trisulfide (TiS_3_), the material under consideration in this study. Differences in the Ti-S bond length along the a (2.65 Å) and b (2.45 Å) axes lead to highly conducting 1D chains along the b-axis and results in strong anisotropic properties^15^,^16^. The red dashed lines mark the titanium, 1D chains in Fig. 1 which are also covalently bonded along the a-axis forming sheets that interact by van der Waals forces. Recent ab initio calculations predict an upper limit for b-axis electron mobilities of more than 10,000 cm^2^/Vs (higher than MoS_2_^17^) and a-axis mobilities of more than an order of magnitude less^15^. To date, TiS_3_ has been exfoliated down to a single-layer (0.7 nm thick) and field-effect transistors and photodetectors have been demonstrated^18^,^19^,^20^,^21^ but a handling of the in-plane anisotropic properties of 2D, few-layer flakes is still lacking.

Here, specifically, we study the in-plane anisotropy of the electrical and optical properties of few-layer TiS_3_ flakes. Electrical transport, angle resolved polarization Raman spectroscopy, and optical transmission measurements are used to probe the anisotropic behavior of thin TiS_3_ specimens. We find that the electrical conductivity shows a marked anisotropy of *G*_max_/*G*_min_ = 2.1 at room temperature and can reach *G*_max_/*G*_min_ = 4.4 at low temperatures. We also note that the Raman spectra of thin flakes show strong in-plane anisotropy that can be used to identify the crystalline orientation of 2D, few-layer TiS_3_ samples. Interestingly, TiS_3_ displays strong linear dichroism, optical absorption that depends on the relative orientation between the materials lattice and incident linearly polarized light, with a magnitude much greater than that observed for BP and other anisotropic 2D materials. Finally, to better understand these findings we calculate the absorption and transmittance spectra in the random-phase-approximation (RPA)^22^,^23^,^24^ and find that the calculations are in qualitative agreement with our experimental findings.

## Results and Discussion

**Figure 2:**
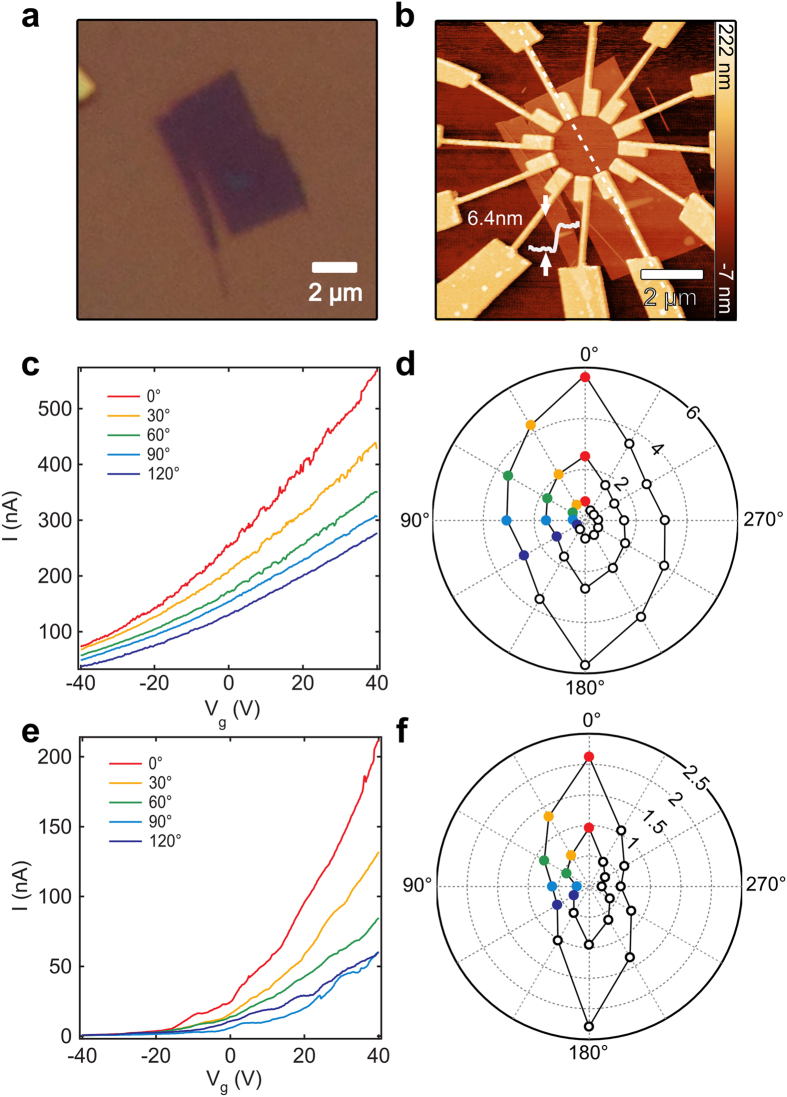
(**a**) Optical image of an exfoliated nanosheet. (**b**) AFM scan of the same nanosheet after patterning 12 Au/Ti electrodes. (**c**) Transfer curves measured at room temperature between 5 pairs of electrodes where 0° is designated as the high conductance (b-axis). (**d**) Polar plot of the room temperature conductance (μS) measured for all 12 pairs of electrodes at back gate voltages of −40 V, 0 V, and 40 V. (**e**) Transfer curves for the same devices at a temperature of 25 K. (**f**) Polar plot of the conductance (μS) at a temperature of 25 K and gate voltages of 40 V (outer curve) and 20 V (inner curve).

TiS_3_ samples are prepared by mechanical exfoliation of bulk TiS_3_ material which is synthesized by sulphuration of titanium disks (as reported in ref. 25). Recently, we have shown that by varying the temperature of the growth process, control over the morphology of the TiS_3_ bulk material can be achieved^19^. At a growth temperature of 400 °C the material grows in a sheet-like morphology permitting the isolation of few-layer, 2D flakes while at 500 °C it grows adopting a ribbon-like morphology, characteristic of the MX_3_ chalcogenides (M = Ti, Zr, Hf and X = S, Se, Te), with high aspect ratio. Figure 2(a) shows an optical image of a 6.4 nm (~9 layers) thick TiS_3_ nanosheet prepared by mechanical exfoliation of the material grown at 400 °C. In order to study the in-plane anisotropic electrical properties of the exfoliated flakes, 12 electrodes are created (using standard e-beam lithography, Ti/Au evaporation, and lift-off) to measure the electrical properties along different directions in rotational steps of 30 degrees. Figure 2(b) shows an AFM scan of the final device with 12 patterned electrodes.

The reduced thickness of the isolated TiS_3_ nanosheet allows us to study the electrical transport in a field-effect geometry, using the underlying 285 nm SiO_2_ as a gate dielectric and the heavily doped silicon as a gate electrode. We modulate the density of the charge carriers by applying a back gate voltage which makes it possible to estimate the field effect carrier mobility. Figure 2(c) shows transistor transfer curves (source-drain current acquired at fixed bias, 100 mV, while sweeping the back gate voltage) measured between two opposing electrodes. We set 0° (dashed white line in Fig. 2(b)) as the high conductance axis and plot the transfer curves for the next four pairs of electrodes in steps of 30° in a counterclockwise direction (0° to 120°). Along the b-axis, we estimate an electron mobility of 25 cm^2^/Vs from the measured transconductance using the standard linear regime, FET mobility calculation (unadjusted for contact resistance)^26^. Figure 2(d) shows a polar plot with the angular dependence of the conductance at gate voltages of −40 V, 0 V, and 40 V. The conductances for angles 0° through 120° correspond to the transfer curves in Fig. 2(c). The minimum (maximum) conductance directions are determined as the a-axis (b-axis) crystalline directions of the TiS_3_ nanosheets. These directions correspond to a conductance anisotropy of *G*_max_/*G*_min_ = 2.1 as well as an anisotropy in the calculated mobilities of *μ*_b_/*μ*_a_ = 2.3 (see Supporting Information, Figure S1 for a polar plot). The anisotropy in the conductance and mobility represents a more than 30% increase over reported anisotropy values for BP FET devices^10^,^27^,^28^. Furthermore, BP shows little change in its anisotropy with decreased temperature whereas we find that the anisotropy in TiS_3_ is strongly temperature dependent^10^. In Fig. 2(e,f) we plot the transfer curves and angle dependent conductances for the same device measured at a temperature of 25 K. The anisotropy in the mobility increases to *μ*_b_/*μ*_a_ = (23 cm^2^/Vs)/(3 cm^2^/Vs) = 7.6 (see supplement figure S1 for polar plots) and we calculate an increase of the anisotropy in conductance to *G*_max_/*G*_min_ = 4.4. Anisotropy values for our few-layer flake devices are slightly lower than the corresponding bulk values^29^,^30^. We speculate that current spreading and increased Coulomb interactions are the main causes which lead to a decrease in the anisotropy when compared with bulk values. Full temperature dependent measurements of nanosheets of thicknesses from bulk to few layers would help ascertain these discrepancies.

**Figure 3:**
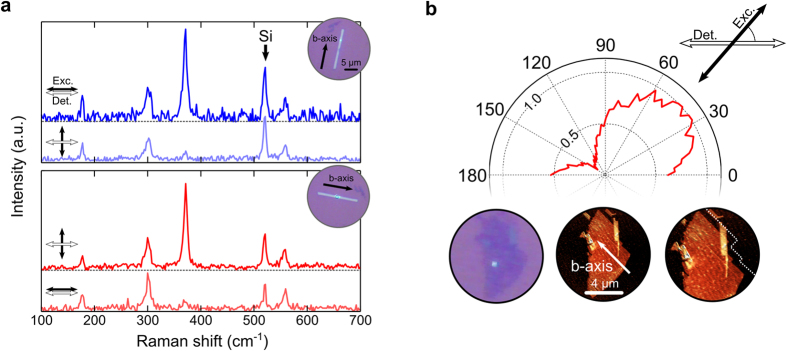
(**a**) Raman spectra of a TiS_3_ ribbon with horizontal excitation and detection polarization (see the arrows in the insets). In the top (bottom) panel the ribbon has been aligned almost perpendicular (parallel) to the excitation/detection polarization. The insets show the position of the TiS_3_ ribbon with respect to the illumination polarization. The peak around 370 cm^−1^ shows the most noticeable change with the change of ribbon alignment. (**b**) Intensity of the 370 cm^−1^ Raman peak of a 3 nm thick TiS_3_ flake (3–4 layers) as a function of the excitation polarization angle (the detection polarization is fixed along the horizontal axis). The minimum intensity occurs when the excitation polarization is parallel to the b-axis of the flake. (Bottom panels) optical and atomic force microscopy images of the studied flake. The determined b-axis is in good agreement with the straight edges of the TiS_3_ flake.

We further characterize the anisotropy of TiS_3_ by employing angle-resolved polarization Raman spectroscopy, as it has proven to be a very powerful tool to delineate the structural and vibrational properties of 2D materials^31^,^32^,^33^. Figure 3 shows Raman spectra acquired on a TiS_3_ ribbon grown at 500 °C under different polarization conditions. Note that we first select a nanoribbon to directly identify the crystalline b-axis which is the preferential growth axis. Fig. 3(a) shows 5 prominent peaks in the Raman spectra of an isolated nanoribbon sample exfoliated on to a Si/SiO_2_ substrate. While the peak at ~520 cm^−1^ is due to a Raman mode of the underlying silicon substrate, the other 4 peaks, (occurring at 177 cm^−1^, 300 cm^−1^, 371 cm^−1^, and 559 cm^−1^) correspond to A_g_-type Raman modes of the TiS_3_ crystal and are in good agreement with the modes reported for bulk TiS_3_^34^,^35^. While all the modes change in intensity with polarization angle (see Supporting Information for angle dependence of all modes), we find that the peak around 370 cm^−1^ is the most sensitive to the relative orientation between the b-axis and the polarization of the excitation laser (top panel of Fig. 3(a)). In fact, its intensity is strongly reduced when the polarization of the excitation is parallel to the b-axis. This is evident after rotating the substrate by ~90° and taking a second Raman spectra measurement with the sample now nearly parallel to the excitation/detection polarization (see bottom panel of Fig. 3(a)). This finding allows us to determine the crystalline orientation of exfoliated TiS_3_ nanosheets whose b-axis direction cannot be distinguished at a glance from the material morphology like in the case of the nanoribbons.

In Fig. 3(b), the intensity of the 370 cm^−1^ Raman peak (measured on a 3 nm thick, about 4 layer thick TiS_3_ nanosheet) is displayed at different excitation polarization angles while keeping the detection polarization parallel to the horizontal axis (see Supporting Information for a direct comparison between Raman spectra for the nanoribbon in Fig. 3(a) and the nanosheet in Fig. 3(b) showing the same Ag-type modes). The minimum of the Raman peak intensity is reached when the excitations polarization forms an angle of 130° with the horizontal axis, indicating the direction of the b-axis in the thin TiS_3_ nanosheet. Below the polar plot, Fig. 3(b) also displays an optical image of the studied flake and an AFM image shown with the same orientation as the optical image. A zoom-in of the AFM image shows how the determined b-axis direction is parallel to the straight edges of the TiS_3_ nanosheet. In Figure S2, the reader will find polar plots, similar to Fig. 3(b), for a TiS_3_ nanoribbon and a TiS_3_ nanosheet acquired at different sample orientation angles showing that the minimum occurs when the polarization of the excitation is aligned with the b-axis.

**Figure 4:**
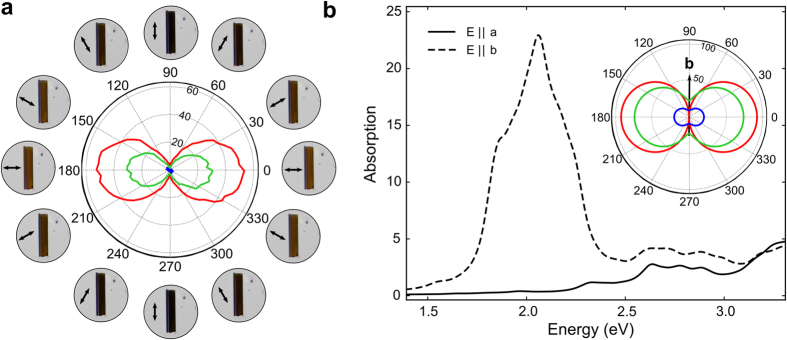
(**a**) Transmittance of the red, green, and blue channels as a function of the excitation polarization angle. (**b**) Calculated absorption spectra when the field is aligned parallel to the b-axis (dashed line) and a-axis (solid line). The inset shows the transmittance in the a–b plane for energies red (1.9 eV), green (2.4 eV), and blue (2.72 eV) excitations.

The anisotropy in the optical properties of TiS_3_ is further characterized using transmission mode optical microscopy. A linear polarizer is placed between the microscope light source and the condenser lens. Transmission mode images are acquired while the polarizer is rotated in steps of ~3°. The transmission is then calculated by normalizing the intensity measured on the TiS_3_ by the intensity measured on the nearby bare substrate. Figure 4(a) shows a polar plot with the angular dependence of the transmission of a TiS_3_ nanosheet. Besides the angle values, Fig. 4(a) also displays the acquired optical images, highlighting the orientation of the excitation polarization with respect to the nanosheet. The polar-plot clearly shows a marked linear dichroism i.e., a variation in the optical absorption for different polarization angles, for the TiS_3_ nanosheet. The transmission reaches a minimum value when the excitation light is polarized along the elongated side of the flake, which corresponds to the b-axis. These absorption characteristics are analogous to that of a wire grid polarizer. The high conducting 1D chains of the TiS_3_ absorb light with a polarization that is parallel to the chain axis (b-axis). The reader can find similar measurements made on a nanoribbon, where the b-axis can be easily determined in the Supporting Information (see Figure S3). The ratio between the b-axis and the a-axis transmission can reach values as high as 30 and decreases for thinner flakes (see Supporting Information, Figure S4). For a direct comparison, we measure the transmission of multilayer BP and MoS_2_ flakes of similar absolute transmission which present transmission ratios of ≈1.4 and ≈1, respectively (see Figure S5 in the Supporting Information).

To better understand and reaffirm the linear dichroic behavior found in the optical transmission measurements, we perform density functional theory (DFT) calculations in combination with many-body techniques (see the Methods section) to calculate the absorption spectrum for bulk TiS_3_. Figure 4(b) shows the calculated absorption spectra for bulk TiS_3_ when the electric field is aligned parallel to the a-axis (black solid curve) and b-axis (black dashed curve). Across the visible wavelengths, the absorption is much larger when the excitation field is parallel to the b-axis than when it is parallel to the a-axis. The inset in Fig. 4(b) shows the calculated transmittance in the a–b plane for red (1.9 eV), green (2.4 eV), and blue (2.72 eV) excitations, agreeing qualitatively well with the experimental transmittance of the red, green, and blue channels in Fig. 4(a).

## Conclusions

In summary, we present electrical and optical measurements of the in-plane anisotropy of a recently isolated 2D material, TiS_3_, with 1D-like properties. From electrical measurements we calculate an anisotropy in the in-plane conductivity of 2.1 at room temperature and 4.4 at a temperature of 25 K. Through Raman characterization, we find that the Raman mode at 370 cm^−1^ is sensitive to the orientation of the b-axis relative to that of the excitation polarization. This allows for simple identification of the high conductance b-axis in TiS_3_ nanosheets. Furthermore, through optical transmission measurements and DFT calculations we show that TiS_3_ exhibits strong linear dichroism where the ratio in the transmission between the b-axis and a-axis reaches values as high as 30. The strong anisotropic properties of TiS_3_ set it apart from the commonly studied 2D materials which have largely isotropic properties and makes it an interesting material for future applications.

### Methods section

#### Synthesis of TiS_3_ bulk material

TiS_3_ has been synthetized by sulfuration of Ti discs (Goodfellow 99.9%, Ø = 10 mm) which had been previously etched in acid mixture (HF:HNO_3_, 4:30 wt%) to clean the surface of any oxide impurities. Sulfuration took place in a vacuum sealed ampoule with sulfur powder (Merk, 99.75%) enough to get the sulfur vapor pressure at 500 °C (∼2 bars) and 400 °C (∼0.5 bars) in order to get nanoribbons and nanosheets, respectively. Time of sulfuration was 20 hours. Method was described in ref. 25.

Fabrication and measurement of FET devices: Few-layer TiS_3_ nanosheets are isolated and exfoliated onto Si/SiO_2_ (285 nm) substrates using a viscoelastic stamp (PDMS). Individual nanosheets are selected by optical color contrast and Ti/Au electrodes are patterned using electron beam lithography, thin-film evaporation, and lift-off in warm acetone. Electrical measurements are performed in a Lakeshore probe station in vacuum.

#### Raman measurements

Raman measurements are performed in a Renishaw system with a 514 nm laser. All spectra are recorded at low powers to prevent laser-induced modification of the samples.

#### Optical Transmission measurements

A *Nikon Eclipse Ci* optical microscope equipped with a *Canon EOS 1200D* digital camera has been employed to perform the optical transmission measurements. A rotation stage with a linear polarizer has been placed between the illumination source and the microscope condenser to control the polarization of the incident light. In order to determine the polarization angle dependence of the transmission, the polarizer has been rotated in 3° steps and a transmission mode image has been acquired at each step. The transmission is then calculated by dividing the intensity transmitted through the bare substrate and that transmitted by the TiS_3_.

#### DFT calculations

Both for Ti and S, the exchange-correlation potential is described self-consistently within the generalized gradient approximation throughout the Perdew–Burke–Ernzerhof’s functional (PBE). For S a norm-conserving Martins–Troulliers’ pseudopotential is used, while for Ti a norm-conserving Goedecker–Hartwigsen–Hutter–Teter’s pseudopotential, including semi-core states for the valence electrons, is used^36^,^37^. We have relaxed the atomic positions and the unit cell vectors with a residual force after relaxation of 0.001 a.u. by starting from the atomic positions provided in ref. 17. The kinetic energy cutoff for the plane wave basis set is 180 Ry. The sampling of the Brillouin zone is 10 × 10 × 10 according to the Monkhorst–Pack scheme. First-principles electronic structure calculations and structure optimisation have been performed with the PWSCF code of the Quantum-ESPRESSO package^38^. As DFT tends to underestimate the electronic bandgap, we performed non-self-consistent G_0_W_0_^39^,^40^ calculations in order to get accurate values for the electronic band structure. The local field effects in the screening calculations have been taken into account and we carefully converged the electronic quasi-particle gap. The G_0_W_0_ corrected DFT bands have been used to calculate the absorption spectra in the random-phase approximation (RPA). To construct the kernel in the RPA we have considered 30 valence and 120 conduction bands. The size of the response function has been carefully converged, hence local field effects corresponding to charge oscillations are accurately included. The plane-wave code Yambo^41^ is used to calculate the quasi-particle corrections and the optical properties in the RPA.

## Additional Information

**How to cite this article**: Island, J. O. *et al*. Titanium trisulfide (TiS_3_): a 2D semiconductor with quasi-1D optical and electronic properties. *Sci. Rep*. **6**, 22214; doi: 10.1038/srep22214 (2016).

## Supplementary Material

Supplementary Information

## Figures and Tables

**Figure 1:**
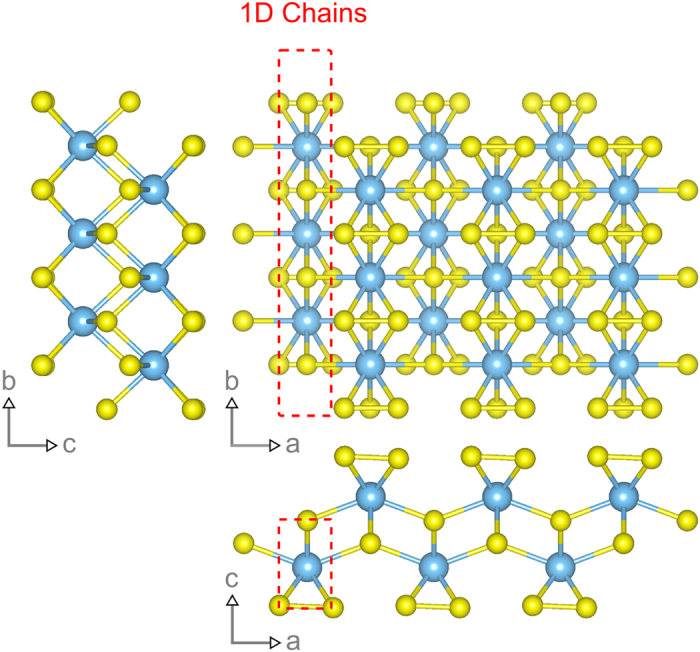
Crystal structure of TiS_3_. The bond lengths between the titanium and sulphur are shorter along the b-axis than along the a-axis. This results in highly conducting chains which lead to strong anisotropic electrical and optical properties. Structure models are produced using VESTA^42^.
