# Supplementary material for: Titanium trisulfide (TiS3): a 2D semiconductor with quasi-1D optical and electronic properties
Source: Sci Rep. 2016 Mar 2;6:22214. doi: 10.1038/srep22214 (PMC4773990; doi:10.1038/srep22214)
Supplement: Supplementary Information [file srep22214-s1.pdf]

Supporting Information

Titanium trisulfide (TiS_3): a 2D semiconductor with quasi-1D optical and electronic properties

Joshua O. Island*, Robert Biele, Mariam Barawi, José M. Clamagirand, José R. Ares, Carlos Sánchez, Herre S.J. van der Zant, Isabel J. Ferrer, Roberto D'Agosta*, and Andres Castellanos-Gomez*

Supporting Information Contents

1. Room temperature and low temperature field effect mobility
   Figure S1: Polar plot of the field effect mobility.
2. Raman polarization dependence and direct comparison between nanoribbons and nanosheets
   Figure S2: Polar plots and Raman spectra for nanoribbons and nanosheets
3. Polar plots of the I3 Raman peak with sample rotation
   Figure S3: Optical images of exfoliated nanoribbon and nanosheet samples with corresponding polar plots of the normalized I3 Raman peak.
4. Polar plots of the transmittance with sample rotation
   Figure S4: Polar plots with corresponding optical images for four sample rotations.
5. Thickness dependence of the angular dependent transmittance
   Figure S5: Polar plots of the transmittance for different thicknesses.
6. Comparison of the angular dependent transmittance for TiS_3, BP, and MoS_2
   Figure S6: Polar plots of the transmittance for TiS_3, BP, and MoS_2.

1. Room temperature and low temperature field effect mobility

The field effect mobility is estimated using the calculated transconductance (dI/dVg) as described in the main text. Figure S1(a) shows a polar plot of the mobility for each pair of electrodes for the device shown in Figure 2(b). The b-axis mobility reaches 25 cm^2/Vs and the minimum mobility is at 120° with a value of 11 cm^2/Vs. The anisotropy ratio is then 2.3 for the room temperature measurements. Figure S1(b) shows the same polar plot oft he mobility at a measurement temperature of 25 K. The ratio increases to μ_b/μ_a = 23 cm^2/Vs / 3 cm^2/Vs = 7.6.

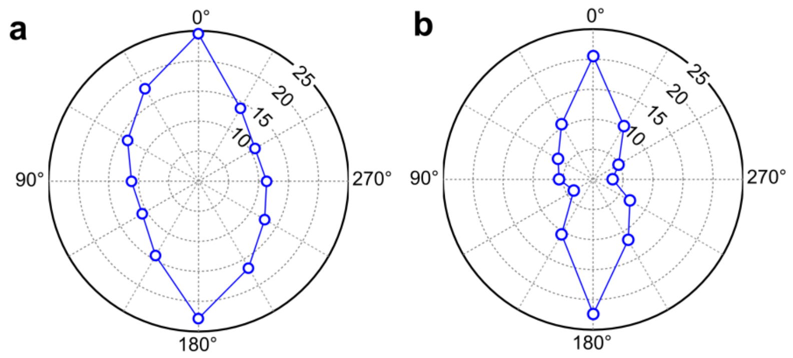

Figure S1: (a) Polar plot of the estimated FET mobility at room temperature. (b) Polar plot of the estimated FET mobility at 25 K.

2. Raman polarization dependence and direct comparison between nanoribbons and nanosheets

In Figure S2(a) we show the angle dependence of all the Raman modes for the nanoribbon and nanosheet in Figure 3 of the main text. While all the modes show some dependence, the peak at 370 cm-1 (blue curve) is most apparent. In Figure S2(b) we directly compare the Raman spectra for the nanoribbon and nanosheet presented in Figure 3 of the main text. This shows that both samples, grown at different temperature, present the same Raman spectra.

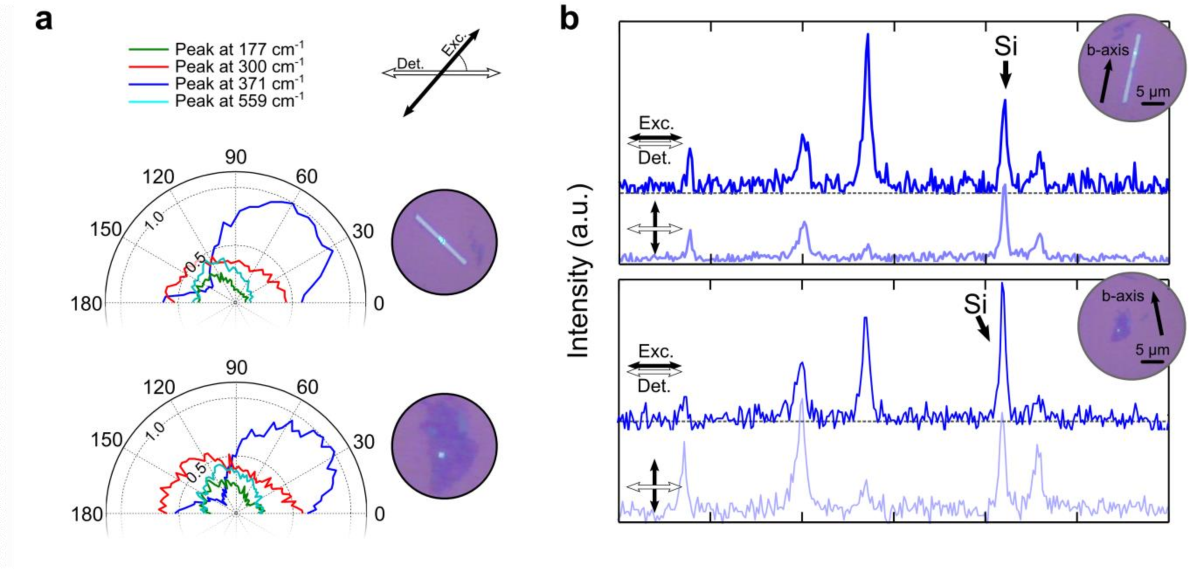

Figure S2: (a) Intensity of all the Raman modes shown in Figure 3 as a function of the excitation polarization angle (the detection polarization is fixed along the horizontal axis). The minimum intensity occurs when the excitation polarization is parallel to the b-axis of the flake. The top panel shows the results for the nanoribbon in Figure 3(a) and the bottom panel shows the results for the nanosheet in Figure 3(b) of the main text. (b) Raman spectra of a TiS_3 ribbon with horizontal excitation and detection polarization (see the arrows in the insets) and for a nanosheet (bottom panel). The peak around 370 cm^{-1} shows the most noticeable change with the change of ribbon alignment.

3. Polar plots of the I3 Raman peak with sample rotation

Here we show that there exists a direct correlation between the minimum of the I3 Raman peak and the b-axis of exfoliated TiS_3 samples by rotating the sample. First we select a nanoribbon which allows us to clearly identify the b-axis which is the preferential growth direction for MX_3 chalcogenides. Figure S3(a) shows optical images of an exfoliated nanoribbon sample and corresponding polar plots of the normalized intensity of the I3 Raman peak (370 cm^{-1}) as the angle between the excitation and detection polarizations are changed. It can be seen that the minimum of the I3 peak is achieved when the excitation polarization is parallel with the b-axis of the nanoribbon. With this in mind, the b-axis of exfoliated few-layer TiS_3 nanosheet samples (grown at 400 C) can be easily determined. Figure S3(b) shows

the same data set for an exfoliated nanosheet. The b-axis for this sample is found to be along the edge of the flake marked with a black line in the optical images.

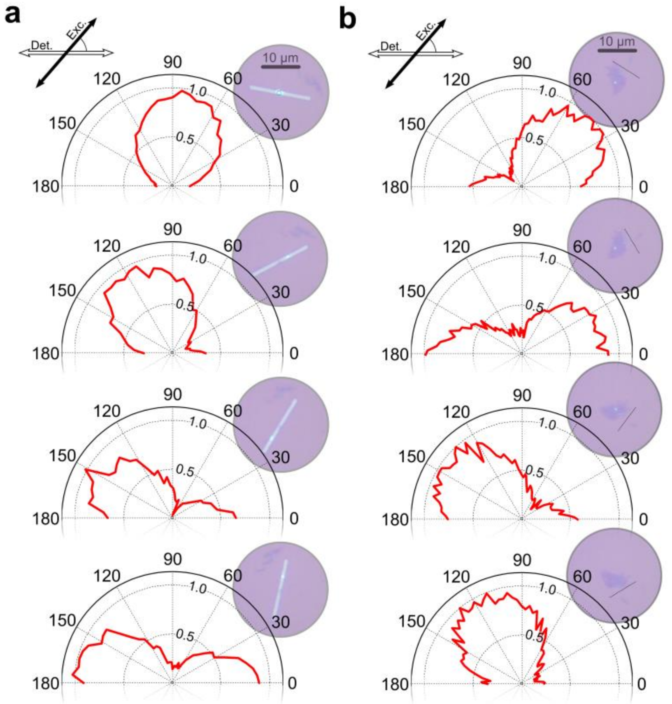

Figure S3: (a) Normalized intensity of the I3 Raman peak of a TiS_3 ribbon as a function of the angle between the excitation and detection polarization. The ribbon has been rotated and the measurement has been repeated several times to illustrate that the minimum of the normalized I3 peak is reached when the excitation polarization is parallel to the TiS_3 b-axis. (b) Normalized intensity of the I3 Raman peak of a TiS_3 nanosheet as a function of the angle between the excitation and detection polarization. The nanosheet has been rotated and the measurement has been repeated several times to illustrate that minimum of the normalized I3 peak is reached when the excitation polarization is parallel to the TiS_3 b-axis.

4. Polar plots of the transmittance with sample rotation

Figure S4 shows that the polar dependence of the transmittance follows subsequent rotations of the sample. The minimum of the transmittance is found to correspond with the b-axis. As the sample is rotated, the corresponding polar dependences rotates as well.

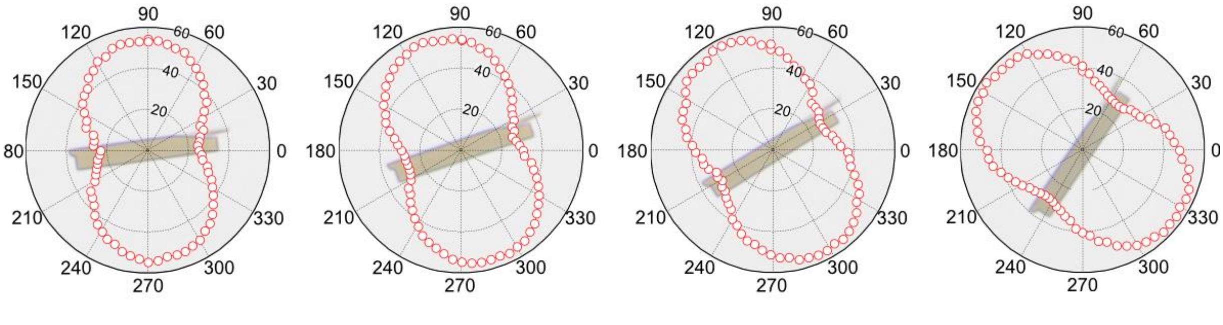

Figure S4. Transmittance as a function of the excitation polarization angle for a TiS_3 wide ribbon, rotated at different angles. The angular dependence of the transmittance follows the rotation of the flake.

5. Thickness dependence of the angular dependent transmittance

Figure S5(a-d) shows the angular dependence of the transmittance for decreasing thicknesses of nanosheet samples. The linear dichroism becomes weaker for thinner samples. This can be directly appreciated in Figure S4(e) where we plot the ratio of the maximum and minimum transmittance as a function of the minimum transmittance (thickness). The ratio decreases for thinner samples.

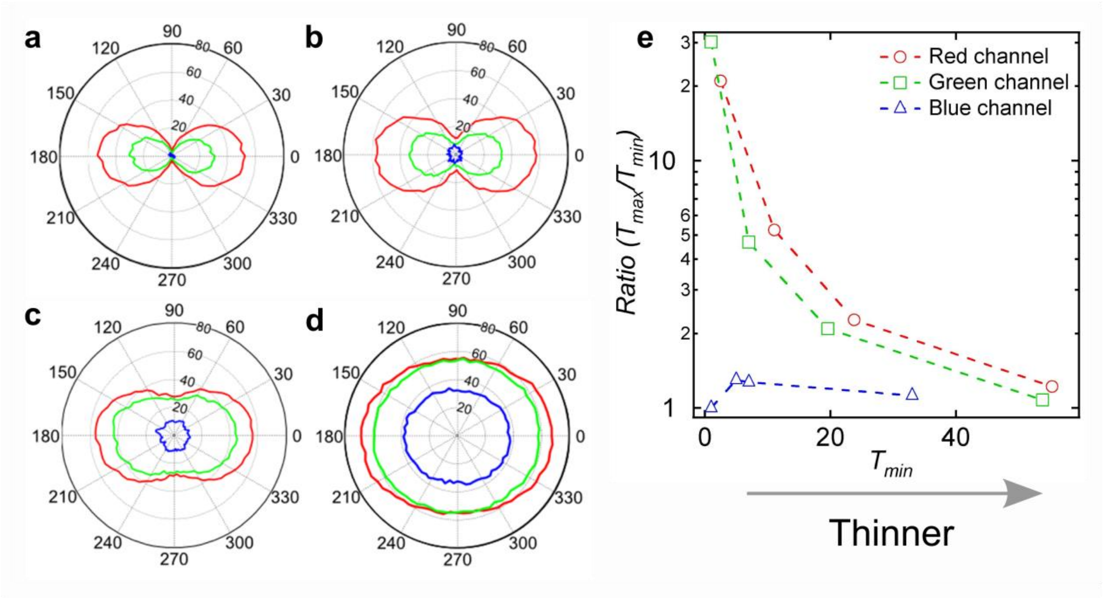

Figure S5. Transmittance measured from the red, green and blue channel of the camera, measured for TiS_3 samples with different thicknesses (from thicker to thinner).

6. Comparison of the angular dependent transmittance for TiS_3, BP, and MoS_2

Figure S6 shows polar plots of the transmittance as a function of excitation angle for samples of TiS_3, BP, and MoS_2 having comparable overall transmittance. It can be seen that the TiS_3 sample has the strongest modulation of the transmittance with a b-axis to a-axis ratio of 30 compared with a ratio of 1.4 for BP. MoS_2 shows little modulation as expected.

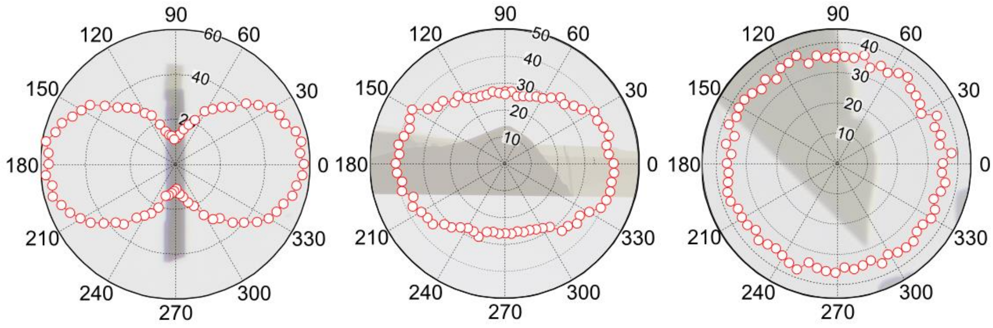

Figure S6. Comparison between the angular dependent transmittance of TiS_3 (left), BP (middle) and MoS_2 (right).
